# Systemic administration of dendrimer N‐acetyl cysteine improves outcomes and survival following cardiac arrest

**DOI:** 10.1002/btm2.10259

**Published:** 2021-10-13

**Authors:** Hiren R. Modi, Qihong Wang, Sarah J. Olmstead, Elizabeth S. Khoury, Nirnath Sah, Yu Guo, Payam Gharibani, Rishi Sharma, Rangaramanujam M. Kannan, Sujatha Kannan, Nitish V. Thakor

**Affiliations:** ^1^ Department of Biomedical Engineering The Johns Hopkins University School of Medicine Baltimore Maryland USA; ^2^ Brain Trauma Neuroprotection Branch, Center for Military Psychiatry and Neuroscience Walter Reed Army Institute of Research (WRAIR) Silver Spring Maryland USA; ^3^ Center for Blood Oxygen Transport and Hemostasis (CBOTH), Department of Pediatrics University of Maryland School of Medicine Baltimore Maryland USA; ^4^ Department of Anesthesiology and Critical Care Medicine Johns Hopkins University School of Medicine Baltimore Maryland USA; ^5^ Department of Neurology The Johns Hopkins University School of Medicine Baltimore Maryland USA; ^6^ Center for Nanomedicine, Department of Ophthalmology Wilmer Eye Institute Johns Hopkins University School of Medicine Baltimore Maryland USA

**Keywords:** cardiac arrest, dendrimer, inflammation, N‐acetyl cysteine, rat

## Abstract

Cardiac arrest (CA), the sudden cessation of effective cardiac pumping function, is still a major clinical problem with a high rate of early and long‐term mortality. Post‐cardiac arrest syndrome (PCAS) may be related to an early systemic inflammatory response leading to exaggerated and sustained neuroinflammation. Therefore, early intervention with targeted drug delivery to attenuate neuroinflammation may greatly improve therapeutic outcomes. Using a clinically relevant asphyxia CA model, we demonstrate that a single (i.p.) dose of dendrimer‐N‐acetylcysteine conjugate (D‐NAC), can target “activated” microglial cells following CA, leading to an improvement in post‐CA survival rate compared to saline (86% vs. 45%). D‐NAC treatment also significantly improved gross neurological score within 4 h of treatment (*p* < 0.05) and continued to show improvement at 48 h (*p* < 0.05). Specifically, there was a substantial impairment in motor responses after CA, which was subsequently improved with D‐NAC treatment (*p* < 0.05). D‐NAC also mitigated hippocampal cell density loss seen post‐CA in the CA1 and CA3 subregions (*p* < 0.001). These results demonstrate that early therapeutic intervention even with a single D‐NAC bolus results in a robust sustainable improvement in long‐term survival, short‐term motor deficits, and neurological recovery. Our current work lays the groundwork for a clinically relevant therapeutic approach to treating post‐CA syndrome.

**Abbreviations**
CA: cardiac arrest
CPR: cardiopulmonary resuscitation
CR: cresyl violet
D‐NAC: dendrimer‐N‐acetylcysteine conjugate
MAP: mean arterial pressure
NDS: neurologic deficit scale
OD: optical density
PCAS: Post‐CA syndrome
ROSC: return of spontaneous circulation
TH: therapeutic hypothermia
TSPO: translocator protein

## 1 INTRODUCTION

Cardiac arrest (CA) accounts for nearly 500,000 deaths annually in the United States and Europe.^1^, ^2^, ^3^, ^4^, ^5^ Survival in patients has continued to increase with improvements in resuscitation care but overall is still low with 50% of cardiopulmonary resuscitation (CPR) attempts restoring spontaneous circulation.^3^, ^5^, ^6^ Despite these advances, outcomes remain bleak in those that survive CA, with irreversible neurologic injury being one of the biggest challenges. Approximately 55% of patients subsequently die after resuscitation,^7^ most commonly related to severe neurological injury.^8^

Neurological injury from CA is multifactorial starting with the primary injury caused by ischemia due to the immediate cessation of cerebral blood flow following CA.^9^ CA‐induced ischemia also secondarily triggers both excitotoxicity and neuroinflammation synergistically to produce microglial activation during the acute period and neuronal cell death.^10^, ^11^, ^12^, ^13^ An increase in inflammatory cytokines and oxidative stress markers is seen as soon as 4 h post‐CA.^14^ Thereafter, reperfusion initiates a secondary injury resulting in a nonspecific peripheral inflammatory response along with inflammation in the brain, perpetuating the microglial activation. This combination of both the primary insult and the secondary reperfusion injury can lead to sustained brain injury resulting in neurocognitive dysfunction and neurofunctional deficits.^9^ Post‐cardiac arrest syndrome (PCAS) is used to describe this unique and complex pathophysiological processes following resuscitation.^15^ We have developed a rodent model of CA that recapitulates many of the key features of human CA and the period following return of spontaneous circulation (ROSC) including the neuroinflammatory cascade seen post‐ROSC in patients.^14^, ^16^, ^17^, ^18^

Currently, targeted temperature management by therapeutic hypothermia (TH) is the standard of care post‐ROSC and has nonspecific effects on inflammation.^7^ A prolonged and sustained elevation of cytokines such as IL‐6 has been associated with worse outcomes and increased severity of PCAS, and TH has not been shown to consistently modify the systemic inflammatory response.^19^, ^20^ Moreover, rewarming after hypothermia has been associated with worsening neuroinflammation and injury in preclinical models,^21^, ^22^ and with impaired cerebral autoregulation in traumatic brain injury and cardiopulmonary bypass.^23^, ^24^ In adults, the rate of death decreases from 55% to 41% with 24 h of TH, however, 30%–40% of survivors continue to have poor neurological outcomes.^7^ While TH is effective, there is a need to adjunct interventions to further improve both survival and neurologic deficits. Notably, elevation of the glial activation marker, 18 kDa translocator protein (TSPO) is observed from 5 to 180 days post‐CA in a rat model of CA.^25^ We hypothesize that more specific mitigation of neuroinflammation by targeting activated microglia will lead to both improvements in survival, neurologic function, and extent of neurologic injury.

Thus, we propose a glia‐targeted approach to mitigate neuroinflammation for the reduction of mortality and neurological impairment post‐CA. Hydroxyl‐terminated poly(amidoamine) (PAMAM) dendrimers are emerging as potential drug delivery platform for neurological indications requiring targeted treatment of glia.^26^, ^27^, ^28^ Dendrimers are taken up selectively by activated microglia and astrocytes in several models of brain injury.^26^, ^27^, ^29^, ^30^ Further, *N*‐acetyl cysteine (NAC) conjugated to hydroxyl‐terminated PAMAM dendrimers (D‐NAC) has been shown to be effective in reducing neuroinflammation and oxidative injury in both large and small animal models at a fraction of the dose of free NAC needed to achieve a therapeutic effect.^27^, ^31^, ^32^ A precursor to glutathione, NAC has both anti‐oxidant and anti‐inflammatory properties and is widely used clinically in children and adults.^33^, ^34^, ^35^, ^36^ NAC is a free radical scavenger as well as an NF‐κB inhibitor.^37^ However, NAC has clinical drawbacks as large, repeated doses are needed in order to achieve the bioavailability necessary for therapeutic effect and high doses of NAC have been shown to increase extracellular glutamate release and can result in neurotoxicity.^28^, ^38^

Here, we explore D‐NAC as a treatment for PCAS. Rats resuscitated after asphyxial‐CA were treated with D‐NAC, or saline, and survival, neurological deficits, and hippocampal damage were evaluated. We demonstrate that a single dose of D‐NAC after ROSC in a rodent CA model improves survival rate and neurological outcomes compared with saline group. Our results also indicate that D‐NAC decreases mortality and improves both brain and behavioral outcomes.

## 2 RESULTS

### 2.1 Systemically administered dendrimer co‐localizes with activated microglia following CA

**FIGURE 1:**
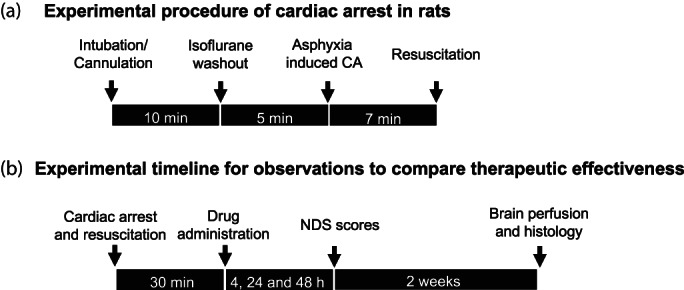
Schematic diagrams of study design. (a) Timeline for cardiac arrest procedure in rats optimized to consistently generate clinically relevant cardiac arrest phenotypes. (b) Sequential outcome measurements collected at different time intervals for comparative effectiveness of therapeutic interventions

**FIGURE 2:**
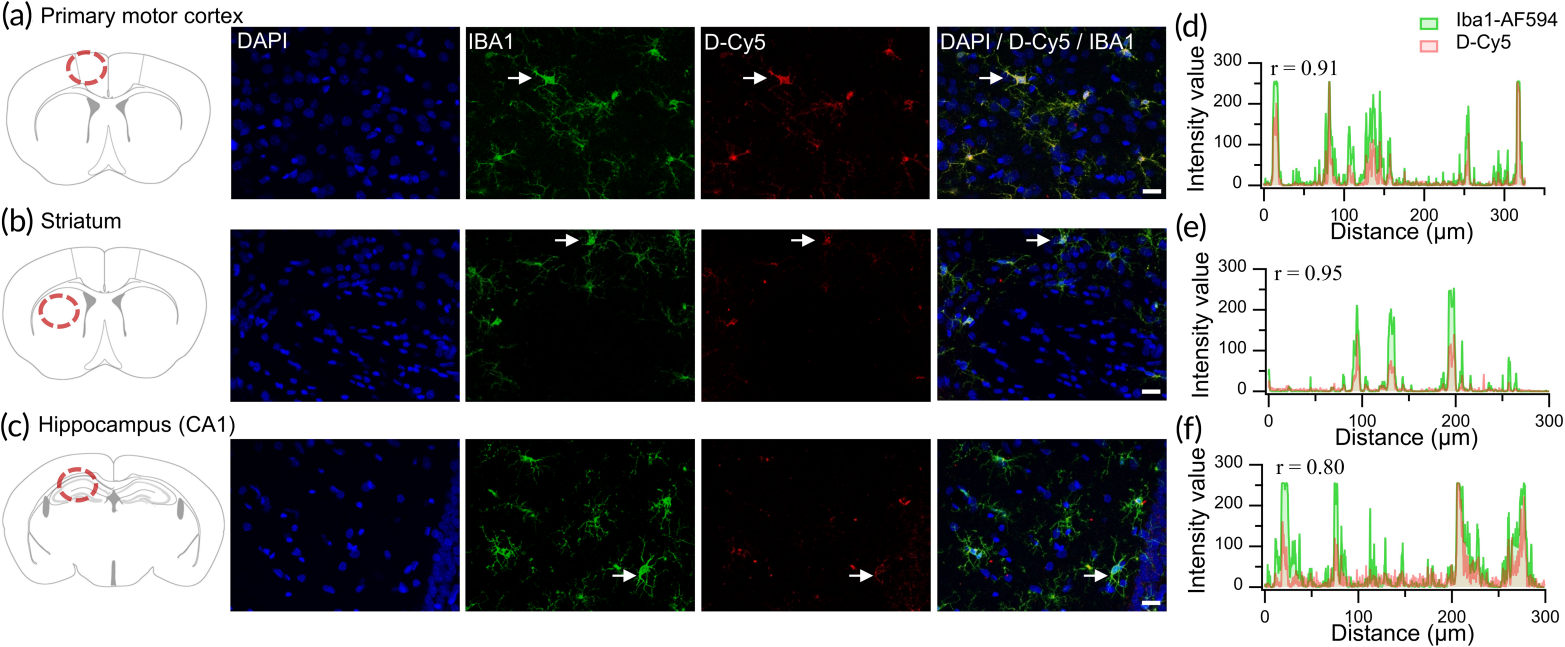
Dendrimer uptake in the primary motor cortex, striatum, and hippocampus. Schematic representation of the anatomical position of images. Representative images (40×) of dendrimer (red) co‐localized with Iba1 stained microglia (green) and DAPI (blue) stained cells in the (a) primary motor cortex, (b) striatum and (c) CA1 region of hippocampus. White arrows denote colocalization. Plot profile of immunofluorescence images acquired from (d) primary motor cortex, (e) striatum and (f) hippocampus. The “*r*” values indicate the Pearson correlation coefficients calculated for respective profile plots. Scale bars: 20 μm

Experimental procedures for CA are as described in Figure 1(a). To demonstrate dendrimer uptake in the brain following CA, dendrimer labeled with Cy5 fluorophore (D‐Cy5) was injected intravenously after 30 min following CA. Rats were sacrificed 4 h after dendrimer administration and brains perfused and fixed for immunohistochemistry. Fixed, cryoprotected and sectioned brains were colabeled with Iba1 and DAPI for microglia and nuclear stain, respectively. D‐Cy5 was found to colocalize with activated microglia in the hippocampus, striatum and primary motor cortex at 4 h following CA. Based on the Pearson correlation and plot profile analysis, the D‐Cy5 signal showed good colocalization with the microglia marker, Iba1, in the motor cortex, striatum, and hippocampus (Pearson correlation coefficient, *r* ≥ 0.80, Figure 2(d), (e), (f)). These results suggest that dendrimers are taken up in the brain post‐CA and selectively localize in the activated microglia (Figure 2).

### 2.2 Systemic D‐NAC improves survival rate

**FIGURE 3:**
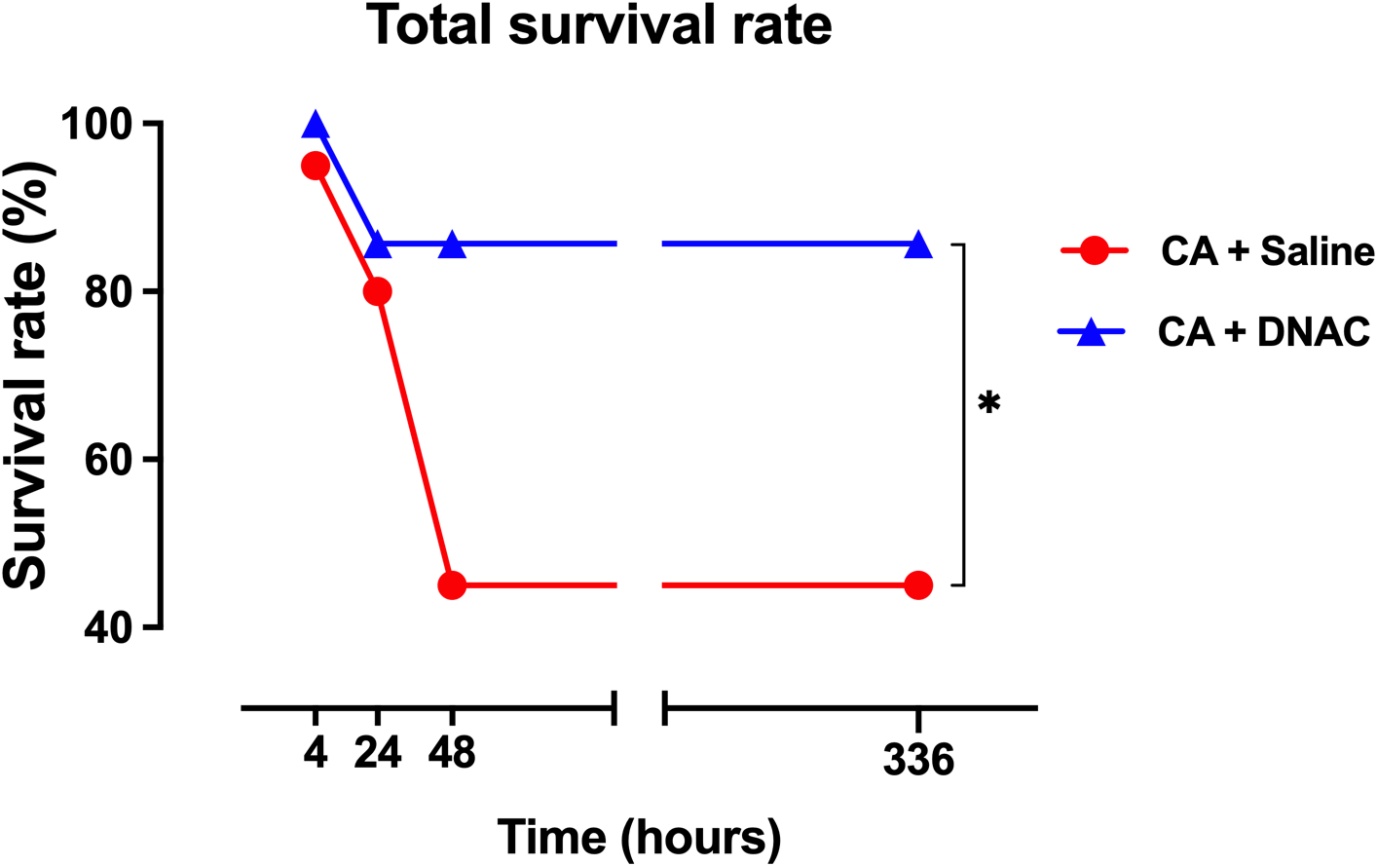
Systemic D‐NAC improves survival from cardiac arrest. Post‐CA treatment with D‐NAC resulted in significant improvements in the survival rate (86%) compared to (45%) in saline‐treated animals (CA + Saline, *n* = 19; CA + D‐NAC group, *n* = 14, *p < 0.05). Fisher's Exact test was conducted. *p < 0.05, compared to saline‐treated animals

A survival analysis was conducted to evaluate D‐NAC effect over a period of 2 weeks. Mortality after resuscitation from CA following initial ROSC was high at 45% (a percentage that is similar to what is seen clinically) with the highest rate of death occurring in the first 48 h after ROSC (Figure 3). In the present study, we observed a significantly improved survival rate after 10 mg/kg of D‐NAC treatment compared to saline‐treated CA rats (86% survival vs. 45%, Figure 3). This suggests that D‐NAC treatment significantly improves post‐ROSC survival.

### 2.3 D‐NAC treatment improves neurologic deficit score

**FIGURE 4:**
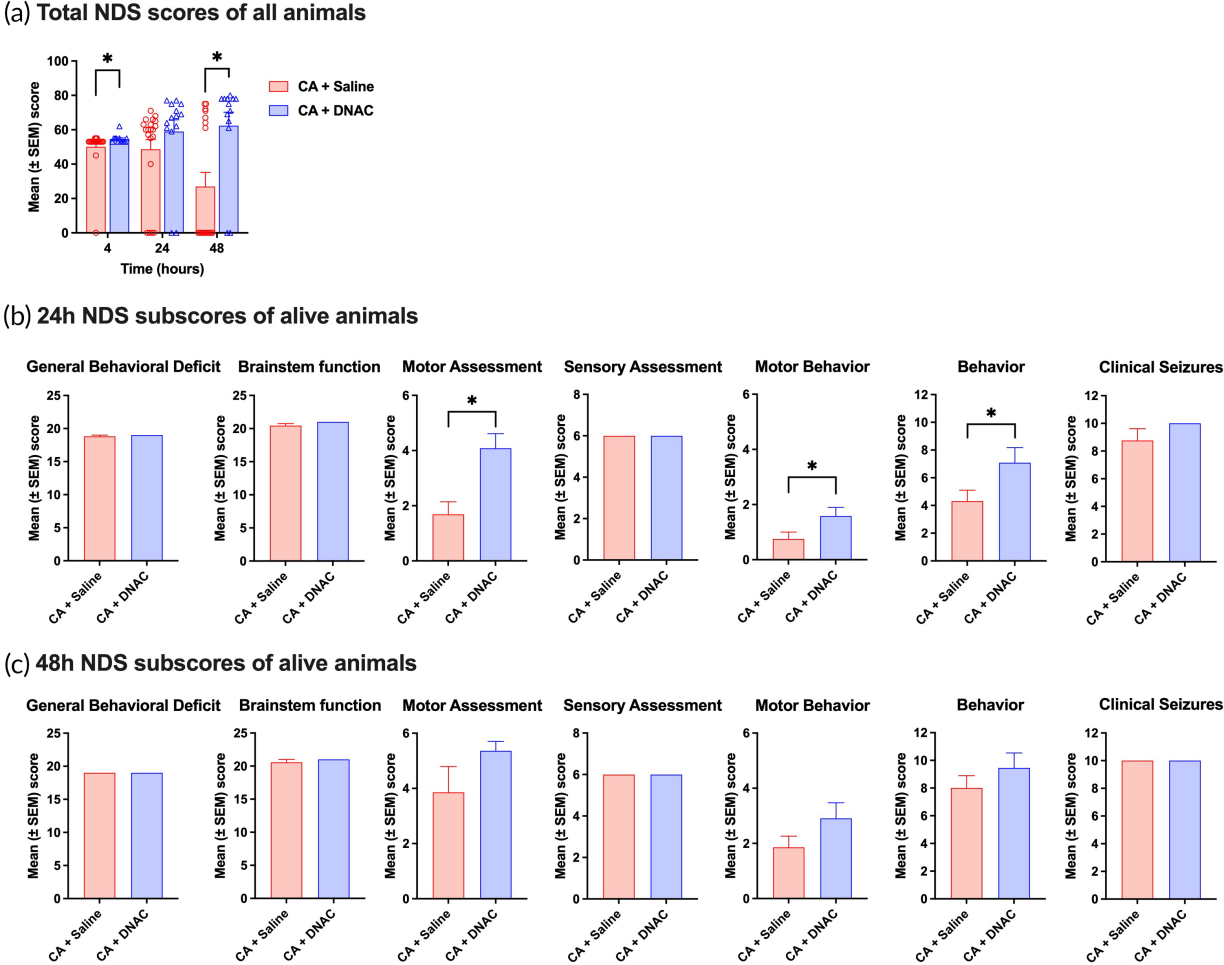
Systemic D‐NAC treatment improves neurologic deficit scores in CA rats. (a) NDS score was increased significantly in CA + D‐NAC group compared to CA + Saline at 4 h (CA + Saline, *n* = 19; CA + D‐NAC, *n* = 14; *p* = 0.007) and 48 h (CA + Saline, *n* = 19; CA + D‐NAC, *n* = 14; *p* = 0.04). (b) NDS subscores at 24 h revealed significant improvement in motor related functions with D‐NAC therapy (CA + Saline, *n* = 16; CA + D‐NAC group, *n* = 12; *p* = 0.002 in motor assessment, *p* = 0.02 in motor behavior, and *p* = 0.04 in behavior, respectively). (c) Comparison of NDS subscores at 48 h with surviving saline‐treated animals indicates a trend in improvement in motor assessments, albeit statistically nonsignificant (CA + Saline, *n* = 7; CA + D‐NAC, *n* = 11; *p* = 0.2). *p < 0.05, compared to saline‐treated animals

Neurological Deficit Scale (NDS) testing is designed to reflect a full range of behavioral repertoire from arousal to coma.^14^, ^17^, ^18^, ^39^ The effect of D‐NAC treatment on NDS scores and subscores were analyzed (Figure 4(a)‐(c)). Gross neurologic testing of rats was conducted at 4, 24, and 48 h. Total NDS is a sum of 18 different tests with various subscores with a maximum of 80 points (best) and a minimum of 0 points (worst) (Figure 4(a), see S1, Supporting Information for scoring scheme). Rats that died were given a score of 0. This was done to account for the high mortality rate and to eliminate the bias of only evaluating the animals who survive. D‐NAC treatment showed significant improvement in total NDS scores within 4 h post‐therapy window (Figure 4(a)). A single systemic dose of D‐NAC therapy resulted in improvement in NDS scores over the studied time course of 4–48 h. At 4, 24, and 48 h post‐CA, D‐NAC‐treated rats showed improved (higher) composite NDS score compared to saline‐treated rats (4 h: *p* = 0.006; 48 h: *p* = 0.04; see Videos S1–S4 at 4 and 24 h). According to our previous study, an NDS score of 60 was utilized as a standard for separating favorable and unfavorable neurologic outcomes after resuscitation.^39^ Based on this prior work, animals with scores above 60 (favorable outcomes) were classified as mild injury phenotype. As shown in Table 1, in CA + D‐NAC group 79% animals were categorized as mild injury phenotype when measured at 24 h, which was significantly higher than the 40% in CA + Saline group (*p* = 0.04). Similarly, a significant difference between CA + D‐NAC group and CA + Saline group was noticed at 48 h following resuscitation (85% vs. 39%, with mild injury, *p* = 0.03). This indicates that a greater fraction of the survivors had a favorable neurologic outcome as demonstrated by a higher NDS score when treated with D‐NAC.

**TABLE 1:** Injury phenotypes of all animals

Time points	Group	Mild injury	Severe injury	Total	% of mild injury phenotype
24 h post‐ROSC	CA + Saline	8	12	20	40.0
	CA + D‐NAC	11	3	14	78.6^*^
	Total	19	15	34	55.9
48 h post‐ROSC	CA + Saline	7	11	18	38.9
	CA + D‐NAC	11	2	13	84.6^*^
	Total	18	13	31	58.1

*Note*: Percentage of animals with a mild injury phenotype (defined as NDS > 60) was significantly higher in CA + D‐NAC group compared to that in CA + Saline group when assessed at 24 h and 48 h post‐ROSC (79% vs. 40% with *p* = 0.04 and 85% vs. 39% with *p* = 0.03, respectively). Severe injury group include animals that died (NDS = 0).

^*^ *p* < 0.05, compared to saline‐treated animals.

NDS subscores were also analyzed at 24 and 48 h for the surviving rats only (Figure 4(c), (d)). At 24 h there was a significant improvement in the motor function related subscores in CA + D‐NAC group (CA + Saline, *n* = 16; CA + D‐NAC group, *n* = 12; *p* = 0.002 in motor assessment, *p* = 0.02 in motor behavior, and *p* = 0.04 in behavior, respectively; Figure 4(c)). At 48 h, however, there were no differences between the groups (CA + saline, *n* = 7; CA + D‐NAC group, *n* = 11; *p* > 0.1; Figure 4(d)). This is likely due to the higher mortality rate in the CA + Saline group. There is a >50% mortality in saline‐treated CA rats between 24 and 48 h after injury (*N* of surviving rats = 16 at 24 h that decreases to 7 at 48 h) when compared to the D‐NAC‐treated rats at 48 h that only decreases by 8% (*N* = 12 at 24 h that decreases to 11 at 48 h). With the most severely injured rats dying within the first 48 h, the remaining saline‐treated rats that survive likely represent animals that sustained less neurologic injury following CA. These data showed that post‐CA D‐NAC has dual effects, survival and early improvement along with sustainable neurological recovery.

### 2.4 D‐NAC treatment promotes longer‐term cell survival in the hippocampus after CA

**FIGURE 5:**
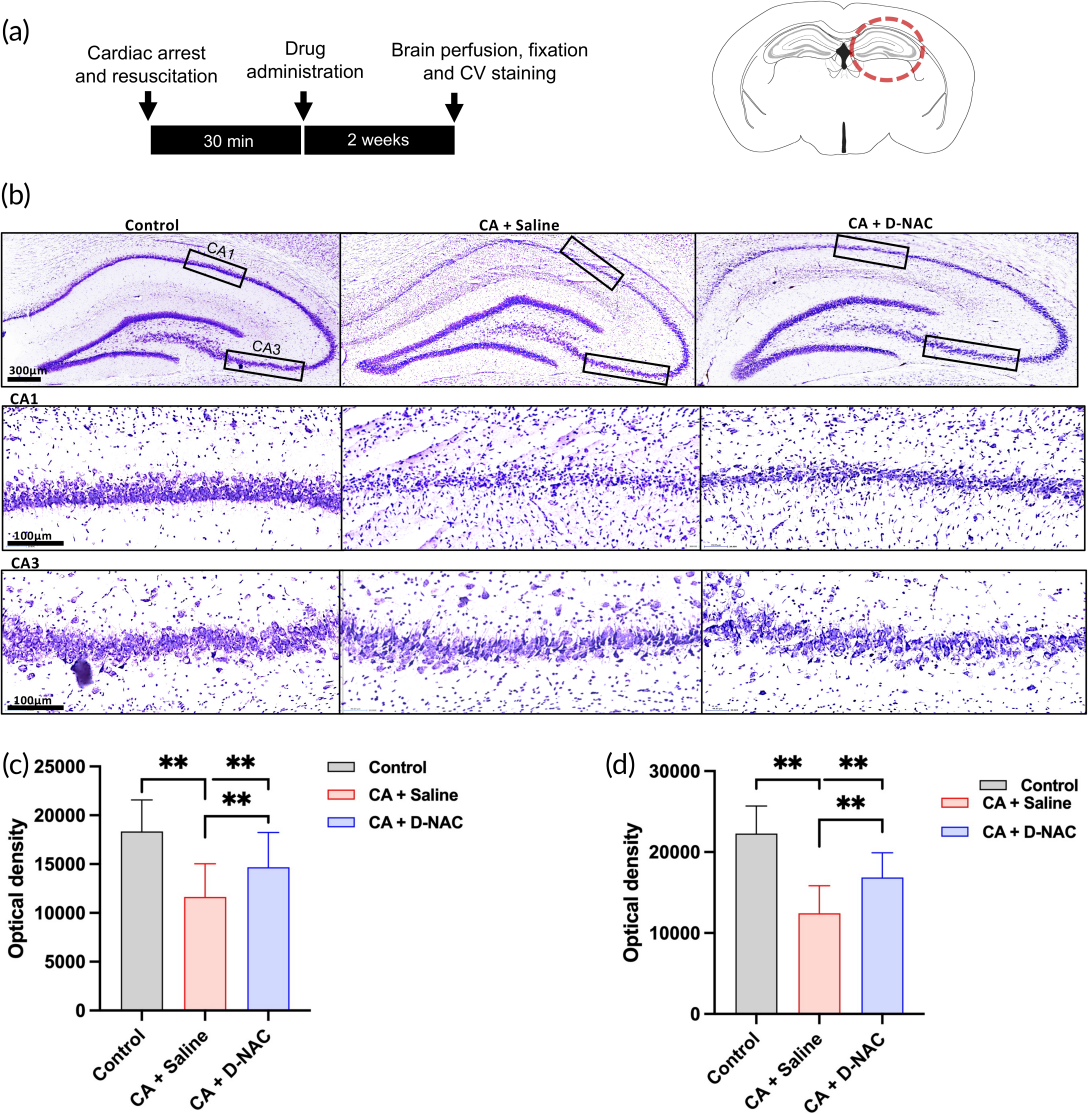
Systemic D‐NAC treatment improves cell survival in hippocampus. (a) Experimental timeline depicting single treatment intervention followed by histological procedures after 2 weeks of CA. Hippocampal sections were stained using cresyl violet. (b) Representative photomicrographs of the hippocampus (upper panel) and the corresponding CA1 and CA3 regions (lower panels) following cresyl violet staining from healthy control, CA + saline and CA + D‐NAC. D‐NAC treatment significantly prevented cardiac arrest induced neuronal cell death in the CA1 and CA3 subfield of the hippocampus, compared to that in the CA + Saline group (c) Optical density measurements (mean ± SEM) from CA1 stratum pyramidale consisting of primarily cell body of projection neurons revealed significant loss after cardiac arrest (healthy control, *n* = 5; CA + Saline, *n* = 6; *p* < 0.001). Single dosage of D‐NAC therapy substantially protected neuronal density (CA + Saline, *n* = 6; CA + D‐NAC, *n* = 7; *p* < 0.001). (d) CA3 stratum pyramidale optical density (mean ± SEM) showed significant decrease in saline‐treated group compared to control (healthy control, *n* = 5; CA + Saline, *n* = 6; *p* < 0.001) that could be recovered to an extent with D‐NAC treatment (CA + Saline, *n* = 6; CA + D‐NAC, *n* = 7; *p* < 0.001). **p < 0.001, compared to saline‐treated animals

Critical brain areas like cortex, striatum, and hippocampus are known to be vulnerable to CA induced injury.^40^, ^41^, ^42^ Therefore, we assessed long‐term histological changes in hippocampus due to CA and the impact of D‐NAC treatment. To this end, we stained and analyzed hippocampal sections from brain perfused 2 weeks (14 days) after CA across different treatment groups (Figure 5(a)). Cresyl violet (CV)‐stained hippocampal sections revealed significant cell loss in CA1 and CA3 stratum pyramidale after CA (Figure 5(b)). Interestingly, a single D‐NAC dose protected neuronal density compared with untreated animals. To quantitate relative density of neuronal population in stratum pyramidale, optical density (OD) measurements were taken and compared across different groups. CA + Saline group showed a significant decrease in OD in both CA1 (healthy control, *n* = 5; CA + Saline, *n* = 6; *p* < 0.001) and CA3 (healthy control, *n* = 5; CA + Saline, *n* = 6; *p* < 0.001) region compared to control. Treatment with a single dose of D‐NAC led to a significant protection of hippocampal neurons in both CA1 (CA + Saline, *n* = 6; CA + D‐NAC, *n* = 7; *p* < 0.001) and CA3 regions (CA + Saline, *n* = 6; CA + D‐NAC, *n* = 7; *p* < 0.001), albeit not bringing it back to the level of healthy controls at this time point.

## 3 DISCUSSION

In these experiments, we evaluated a therapeutic approach to mitigate neurological injury post‐CA via targeted delivery of D‐NAC to activated glia. First, we see that hydroxyl‐terminated PAMAM dendrimer, when given systemically 30 min post‐CA, is successful at localizing in activated glia by 4 h after administration. We see uptake of these dendrimers in microglia in the hippocampus, cortex, and striatum; areas that demonstrate neuroinflammation post‐CA as previously demonstrated by our group in this model.^40^ This also confirms previous findings in other relevant disease models, including a mouse model of neonatal hypoxia‐ischemic encephalopathy and a canine model of hypothermic circulatory arrest demonstrating glial uptake of PAMAM dendrimers in similar regions displaying microglial activation (e.g., hippocampus).^27^, ^31^

Inflammation has been shown to be a key driver in injury post‐ROSC. In patients, this is most prominently seen with exacerbations of systemic inflammation upon rewarming after TH.^21^, ^22^ Recently through the use of animal models of CA, microglia have been implicated in the injury and chronic inflammation observed in the brain post‐CA. TNFα (among other cytokines) increases as soon as 2–3 h after injury in both the serum and the brain.^43^, ^44^ Microglial activation and migration into damaged areas have been observed as early as 3 days post‐CA.^45^ Further, glial activation has also been assessed using a radiolabeled TSPO ligand. TSPO binding overall was increased from 5 to 180 days post‐CA and CA time correlated with hippocampal TSPO binding at all timepoints.^25^ Previously our group has shown that D‐NAC mitigates the microglial response through both in vivo and in vitro studies,^26^, ^27^, ^32^, ^46^, ^47^ and this work extends our results to a clinically relevant rodent model of global ischemic injury following CA.

We also observed that the survival rate is markedly improved in rats receiving dendrimer‐delivered NAC (D‐NAC) (10 mg/kg on a NAC basis) after ROSC. We used an asphyxia time of 7 min to ensure moderate‐to‐severe injury following ROSC.^16^ This led to a 44% survival at 14 days post‐ROSC as observed in the saline‐treated CA animals, with the highest mortality occurring in the first 48 h. This is similar to what is seen clinically, based on the GWTG‐R (Get with the guidelines‐resuscitation) registry where survival for in hospital CA varied between ~20% and 40% in 2017 depending on the cause of CA.^48^ Our model's results mimic the clinical observation that a large proportion of deaths occurred early in the first 24 to 48 h post‐ROSC in patients. We find that D‐NAC treatment 30 min after ROSC resulted in 86% of rats surviving which is nearly double that of the saline‐treated rats. Repeated doses of NAC alone have been shown to cause a decrease in survival in a mouse model of Rett syndrome due to increased extracellular glutamate release via the cystine‐glutamate antiporter (system x_c‐_), while D‐NAC improved survival.^28^ Thus, these and previous data demonstrate that dendrimer‐mediated delivery of NAC to activated glia is essential for this dose of NAC (10 mg/kg) to be effective in promoting survival after CA.

In tandem with an increase in survival, we see improvement in global neurologic function with D‐NAC administration. When accounting for all rats that were treated, D‐NAC‐treated rats showed both early (4 h) and late improvements (48 h) compared to saline‐treated rats. Further evaluation of the NDS subscores in only the live animals reveals that D‐NAC has the most profound improvement on gross motor function specifically at 24 h post‐CA. The likely reason for the lack of statistical significance at 48 h is the high mortality of the more severe CA + Saline rats from 24 to 48 h. With the most severe rats dying, the remaining saline‐treated rats represent a less severe subset of post‐CA rats. The most common pattern of impairments in post‐CA patients is a combination of memory/executive and motor deficits.^49^, ^50^ Our data demonstrate the ability of a single systemic dose of D‐NAC to enhance sustainable clinically relevant neurological outcomes.

Neuroprotection was also observed within the hippocampus following D‐NAC treatment. Significant histological changes were detected in the CA1 and CA3 subregions of the hippocampus of CA rats. Specifically, we detected decreased hippocampal cell density in CA rats compared to healthy controls and found that D‐NAC treatment increased cell density compared to saline‐treated CA rats. These findings align with recent data indicating a role for neuroinflammation and specifically glia in neuronal death post‐ischemia.^51^, ^52^, ^53^ Further, several treatments (including propofol, soluble epoxide hydrolase, and (+)‐naltrexolone) that result in either decreased neuroinflammation or a shift toward alternative activation also show decreased neuronal damage after CA.^44^, ^45^, ^54^, ^55^ Decrease in both global and hippocampal volumes has been reported in patients following CA.^42^, ^56^, ^57^, ^58^

The therapeutic benefit of the current standard of care, hypothermia after CA is low with a number‐needed‐to‐treat of six patients in order to save one life.^7^ The therapeutic limitations are likely due to the time delays to starting treatment. Current guidelines recommend TH in patients that do not regain consciousness post‐ROSC in part because hypothermia is difficult to tolerate if awake.^59^ TH is typically initiated within 2–3 h post‐ROSC, and once the decision to initiate TH is made, it can take up to 4 h to reach target core temperature.^7^ This time delay has proven to be critical in animal models. We have previously shown that hypothermia immediately after CA in this model is more effective in rescuing the electrophysiological function (as measured by the qEEG technique) than conventional hypothermia initiated 6 h post‐CA.^17^ Thus, we conclude that time to deliver treatment is an important factor in rescuing the brain function, but that TH in the clinical setting simply cannot be implemented at this faster time scale. Here we demonstrate a quicker, more effective, and easily tolerated alternative to mitigating neuroinflammatory processes for the reduction of post‐ROSC mortality and neurological injury with a single systemic dose of D‐NAC 30 min after ROSC. D‐NAC treatment was effective in increasing the survival rate, improving gross neurological outcome (total NDS score), and providing neuroprotection in the hippocampus. D‐NAC may also be used in combination with TH to enhance neuroprotection. We have previously demonstrated that hypothermia does not decrease the microglial localization of dendrimers in a model of hypoxia‐ischemia.^27^, ^60^

Long‐term cognitive outcomes are still poor in patients that survive CA and represent a largely unmet clinical need.^8^, ^61^ It is likely that poor neurological outcomes are mediated by persistent neuroinflammation. Chronic neuroinflammation after injury or insult is common and recent reports demonstrate that even after hypothermia, neuroinflammation can persist chronically in patients' post‐CA. Patients with poor neurological outcomes showed greater BBB impairment 3 months post‐CA compared to patients classified as having good neurological outcome suggesting that neuroinflammation in some form persists.^62^ This may be one of the reasons that TH alone is insufficient in reducing neurological deficits.^63^ It may be that low‐grade chronic neuroinflammation persists and negatively impacts cognition and executive function, modalities commonly affected in patients. Future experiments will more systematically evaluate the impact of D‐NAC on chronic neuroinflammation and long‐term cognitive function.

In summary, hydroxyl‐terminated PAMAM dendrimers are able to quickly localize in activated glia after ROSC, and early intervention with D‐NAC leads to improved survival rate, neurological function, and hippocampal neuronal survival. These results demonstrate that hydroxyl‐terminated PAMAM dendrimers appear to be highly viable candidates for fast and glial‐targeted delivery of anti‐inflammatory agents. Having shown the profound improvements in survival 48 h post‐CA, we intend to investigate possible mechanisms by which this is occurring as well as further dose optimization and delivery times for promoting survival, recovery of neurological cognitive/executive function. Future studies would also aim to use both male and female model of CA to explore gender differences in injury and optimization of therapeutic efficacy.

## 4 MATERIALS AND METHODS

### 4.1 Asphyxial‐CA Model

Our previous work^14^, ^16^, ^17^, ^18^, ^39^, ^64^ has established and extensively characterized adult male Wistar rats as a model for studying CA associated injury and therapeutic approaches such a TH. Adult male Wistar rats (Charles River, Wilmington, MA) that are 300–350 g were habituated for 1 week prior to the procedures. The rats had free access to food and water before and after the experiments and were housed in a quiet environment with 12‐h day‐night cycles. All experimental procedures were approved by the Johns Hopkins University Animal Care and Use Committee. The animals were randomly divided in three different groups: sham animals (without CA), CA + Saline treatment, CA + D‐NAC (10 mg/kg on a NAC basis, IP). Animals were subjected to asphyxia‐induced CA (Figure 1(a)). Our previous studies have demonstrated that asphyxic CA for 7 min duration in this model results in significant neuronal injury. The resulting injury is broad and significant but still results in 80% survival (resuscitation) of the animal post CA, making this a clinically relevant model and allowing evaluation of the neuroprotective effect of D‐NAC.^14^, ^16^, ^17^, ^18^, ^39^, ^45^ Our CA protocol has been described in detail previously.^14^, ^16^, ^17^, ^18^, ^39^, ^45^ Briefly, rats were endotracheally intubated by direct laryngoscopy and mechanically ventilated with 2% isoflurane in 50% O_2_ + 50% N_2_ gas, after which the femoral artery and vein were cannulated with polyethylene 50 tubing catheters to monitor blood pressure and sample arterial blood gases and to administer intravenous medications. We carried out 10 min of baseline recording for mean arterial pressure (MAP) under isoflurane. Following baseline recording, anesthesia was washed out for a total 5 min, starting with 2 min of 100% O_2_ without isoflurane to capture nonanesthetized MAP (Figure 1(a)). Then for 3 min, FiO_2_ was decreased to 20% with 80% of N_2_ gas (room air). Rocuronium bromide 2 mg/kg IV was administered for muscle paralysis at 2 min of washout time. Following 5 min of gas washout, global asphyxia was induced by stopping and disconnecting the ventilator and clamping the tracheal tube. Global asphyxia was accompanied by transient hypertension, followed by progressive bradycardia, hypotension and eventual CA (defined by MAP<10 mmHg and nonpulsatile‐pressure wave) are observed (Figure 1(a).^18^

### 4.2 Resuscitation

After the period of 7 min of asphyxia, CPR was initiated by unclamping the tracheal tube, restarting mechanical ventilation with 100% O2, administering epinephrine (5 μg/kg, i.v.), and applying sternal chest compressions with two fingers (~200 compressions/min), to generate MAP > 50 mmHg within 2 min was defined as a successful ROSC and NaHCO3 (1 mmol/kg, i.v.) to normalize the arterial pH. After undertaking the aforementioned measures ROSC were achieved the time was noted. Anesthesia was not provided post‐resuscitation to minimize drug effects on recorded electrical (EEG) signals and to facilitate the return of arousal. Following successful resuscitation, the animal was hyperventilated (TV[the amount of air that will go into the patient's lungs with each breath] 10 mL/kg, RR [respiratory rate] 65/min, and PEEP [positive end expiratory pressure] 3 cm H_2_O) for 10 min. RR was adjusted to 50/min. Rectal temperature was maintained at 37.0°C using a homeothermic blanket systems with flexible rectal temperature probe (Harvard Apparatus, MA, USA). Once a rat achieved spontaneous respiration, it was extubated, vascular catheters were removed, and sutures were placed in the femoral region^8^, ^9^, ^12^, ^38^ (Figure 1(a)).

### 4.3 Dendrimer‐Cy5 for determination of cellular localization of systemically administered dendrimers

A fluorescent chrome (in this case, Cyanine 5; Cy5) was conjugated to generation 4 hydroxyl‐terminated (G4‐OH) PAMAM dendrimers (Dendritech) in‐house and characterized as previously described.^65^ Briefly, G4‐OH dendrimers were partially amine‐functionalized and reacted with Cy5‐NHS ester dye. The purified final conjugate was characterized by proto NMR, HPLC/GPS and fluorescence spectroscopy and fluorescence spectrum pattern was confirmed for Cy5 (excitation: 645 nm; emission: 662 nm). Rats were given intravenous injections of 10 mg/kg of D‐Cy5 (~1/5th the dendrimer amount in D‐NAC 10 mg/kg) dissolved in 0.2 mL of saline 30 min post‐CA resuscitation. The chemical structure of D‐NAC is illustrated in Figure S1. Rats were monitored and evaluated for 4 h after injection, and were subsequently euthanized, perfused transcardially with saline and then brains were fixed for cryosectioning and staining.

### 4.4 Treatment with D‐NAC

D‐NAC was synthesized using generation 4 hydroxyl‐terminated PAMAM dendrimers (Dendritech) as previously described.^66^ We have previously demonstrated reproducible and scalable D‐NAC synthesis that results in a payload of about 22–23 NAC molecules per dendrimer.^66^ At 30 min post‐ROSC, the rats were randomized into two groups to receive (1) saline treatment (CA + Saline control) at 0.2 mL, or (2) D‐NAC in 0.2 mL saline (CA + D‐NAC containing 10 mg/kg on a NAC basis) treatment intra‐peritoneal (i.p.) (Figure 1(b)). The sham group (i.e., healthy control) animals were not subjected to CA or any other manipulations.

### 4.5 Neurologic deficit scale score

The NDS score, which was previously developed and validated extensively, ranges from 0 to 80 and serves as a surrogate quantitative rodent neuro‐deficit and coma score, analogous to human coma scores.^14^, ^17^, ^18^, ^39^ See supplementary information for details of NDS sub‐scores components; Table S1). The NDS was determined at 4, 24, and 48 h post‐ROSC (Figure 4). For the differential between mild and severe injury, score 60 was used as a cut‐off value following our previous publication.^39^ Trained personnel who were blinded to the control and treatment groups conducted NDS examinations.^14^, ^16^, ^17^, ^18^, ^39^, ^64^

### 4.6 Histopathology

Brain was transcardially perfused with 0.1 M phosphate buffer saline followed by 4% paraformaldehyde in 0.1 M phosphate buffer saline (PBS, pH 7.4) and stored overnight in the same solution. The fixed brain was then transferred into cryoprotectant 10% sucrose solution followed by 30% sucrose solution until the brain sunk to the bottom of the vial after transferring each time. The brains were removed and serially cut into 30‐μm thickness of coronal sections in a cryostat (Leica, Germany). The brain sections thus obtained were used for histological analysis. For CV staining, 1.0% (w/v) Cresyl Violet acetate (Sigma, St. Louis, MO, USA) solution was made, and glacial acetic acid (Sigma‐Aldrich) was added to this solution. The sections were washed thoroughly with 0.1 M phosphate buffer saline and stained with CV solution at room temperature and washed with distilled water. The stained sections were dehydrated in serial ethanol (50%, 70%, 80%, 90%, 95%, and 100%) baths. After dehydration, the sections were coverslipped. The hippocampus formation in different treatment groups was digitally scanned using a scanner microscope (Nikon, Tokyo, Janpan). To quantitatively analyze the density of CV‐stained neurons in the CA1 and CA3, five random sections was chosen from each animal and five random spots (200 μm × 200 μm) were selected at 40× magnification on each region from each section. Densities of CV‐stained neurons were evaluated based on an OD after transformation of each image to 8‐bit gray level using Image J software (National Institutes of Health, Bethesda, MD, USA). The background of each slide was subtracted, and the final number was presented as the OD of CV‐stained neurons.

### 4.7 Immunofluorescence

To evaluate the colocalization of D‐Cy5 and microglia, 30 μm coronal brain sections were incubated overnight at 4°C with goat anti‐IBA1 (1:250, Abcam, MA, USA). Sections were subsequently washed and incubated with donkey anti‐goat secondary antibody conjugated with AlexaFluor 594 (1:500; Invitrogen) for 2 h at room temperature. Next, the sections were incubated with DAPI (1:1000, Invitrogen) for 15 min. After wash, the slides were dried and cover slipped with mounting medium (Dako, Carpinteria, CA, USA). Confocal images were acquired with Zeiss ZEN LSM 880 (Zeiss, CA, USA) and processed with Image J software (National Institutes of Health).

### 4.8 Statistics

Data are presented as mean ± SEM. Survival data and injury phenotype were analyzed using discrete chi‐squared or fisher's exact tests at each time point. NDS data were analyzed using two‐way ANOVAs and one‐way ANOVAs. When appropriate, Kruskal–Wallis tests were performed instead of one‐way ANOVAs. Histological data were analyzed using Generalized Estimating Equations via customized scripts. Statistical significance was set at *p* ≤ 0.05 (marked as *) and *p* ≤ 0.001 (marked as **); testing was done with modified Bonferroni correction for multiple comparisons.

## CONFLICT OF INTEREST

Rangaramanujam M. Kannan, Sujatha Kannan, and Rishi Sharma have awarded and pending patents relating to the D‐NAC and dendrimer platform. Rangaramanujam M. Kannan and Sujatha Kannan are co‐founders and have financial interests in Ashvattha Therapeutics Inc., a startup involved with the translation of dendrimer drug delivery platforms.

## AUTHOR CONTRIBUTIONS

**Hiren Modi:** Conceptualization (lead); data curation (lead); formal analysis (equal); methodology (equal); project administration (equal); writing – original draft (equal). **Qihong Wang:** Data curation (equal); investigation (equal); writing – review and editing (equal). **Sarah Olmstead:** Conceptualization (equal); data curation (equal); formal analysis (equal); methodology (equal). **Elizabeth Khoury:** Conceptualization (equal); data curation (equal); formal analysis (equal); methodology (equal); project administration (equal); supervision (equal); validation (equal); writing – original draft (equal); writing – review and editing (equal). **Nirnath Sah:** Conceptualization (equal); data curation (equal); formal analysis (equal); methodology (equal); project administration (equal); resources (equal); supervision (equal); validation (equal); writing – original draft (equal); writing – review and editing (equal). **Yu Guo:** Conceptualization (equal); data curation (equal); formal analysis (equal); methodology (equal); writing – original draft (equal). **Payam Gharibani:** Conceptualization (equal); data curation (equal); formal analysis (equal); methodology (equal); visualization (equal); writing – original draft (equal). **Rishi Sharma:** Investigation (equal); methodology (equal); resources (equal). **Kannan Rangaramanujam:** Conceptualization (equal); funding acquisition (equal); project administration (equal); supervision (equal); visualization (equal); writing – original draft (equal); writing – review and editing (equal). **Sujatha Kannan:** Conceptualization (equal); data curation (equal); formal analysis (equal); funding acquisition (equal); investigation (equal); methodology (equal); project administration (equal); resources (equal); supervision (equal); visualization (equal); writing – original draft (equal); writing – review and editing (equal).

## Supporting information


**Video S1.** CA + D‐NAC—4th post‐ROSC

**Video S2**. CA + D‐NAC—24h post‐ROSC

**Video S3**. CA + Saline—4h post‐ROSC

**Video S4**. CA + Saline—24h post‐ROSC

**Figure S1** The structural representation of dendrimer‐N‐acetyl cysteine (D‐NAC)
**Table S1**. The Neurological Deficit Scale Score (NDS score)

## Data Availability

The data that support the findings of this study are available from the corresponding author upon reasonable request.
