# Supplementary material for: Systemic administration of dendrimer N‐acetyl cysteine improves outcomes and survival following cardiac arrest
Source: Bioeng Transl Med. 2021 Oct 13;7(1):e10259. doi: 10.1002/btm2.10259 (PMC8780014; doi:10.1002/btm2.10259)
Supplement: Supplementary file 5 — Figure S1 The structural representation of dendrimer‐N‐acetyl cysteine (D‐NAC) Table S1. The Neurological Deficit Scale Score (NDS score) [file BTM2-7-e10259-s001.docx]

**Supplementary Materials**

**Figure S1: The structural representation of Dendrimer-N-acetyl cysteine (D-NAC).**

**Table S1: The Neurological Deficit Scale Score (NDS SCORE).**

**
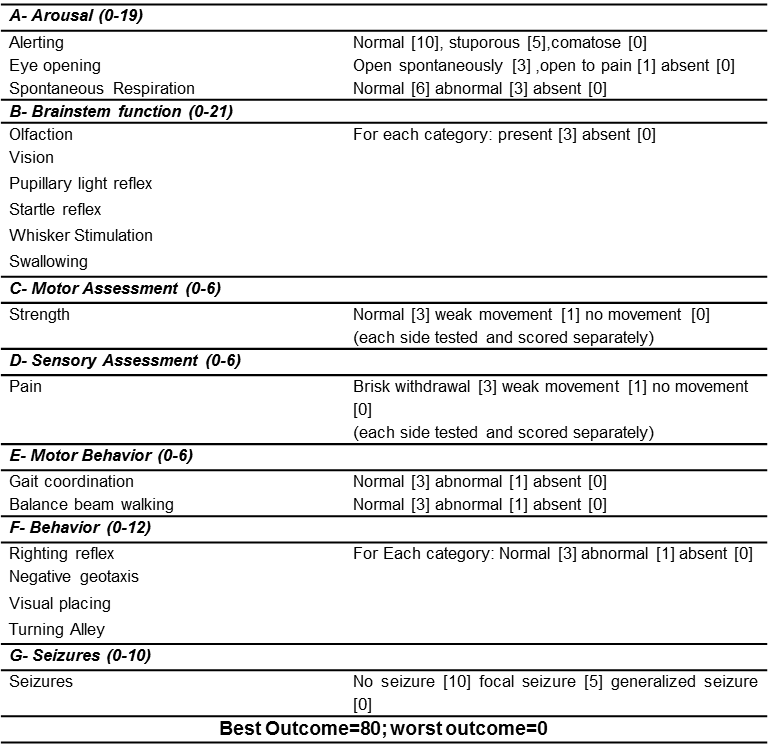
**
